# Cross-Sectional Questionnaire of Donkey Owners and Farriers Regarding Farriery Practices in the Faisalabad Region of Pakistan

**DOI:** 10.3390/ani12060709

**Published:** 2022-03-11

**Authors:** Raja Zabeeh Ullah Khan, Sarah Margaret Rosanowski, Waqar Saleem, Rebecca Sarah Victoria Parkes

**Affiliations:** 1 Department of Clinical Sciences, College of Veterinary Medicine and Biomedical Sciences, Colorado State University, Fort Collins, CO 80521, USA; zabeeh.khan@colostate.edu; 2 Independent Veterinary Epidemiology Consultant, EVC Limited, Hong Kong SAR 999077, China; s.rosanowski@gmail.com; 3 Laboratory of Virology, Faculty of Veterinary Medicine, Ghent University, 9820 Merelbeke, Belgium; waqar.saleem@ugent.be; 4 Department of Veterinary Clinical Sciences, Jockey Club College of Veterinary Medicine and Life Sciences, City University of Hong Kong, Kowloon, Hong Kong SAR 518057, China; 5 Centre for Animal Health and Welfare, Jockey Club College of Veterinary Medicine and Life Sciences, City University of Hong Kong, Kowloon, Hong Kong SAR 518057, China

**Keywords:** donkey, farriery, working equid, Pakistan, client interactions

## Abstract

**Simple Summary:**

Ensuring the welfare of donkeys in low-middle income countries (LMICs) requires a collective approach involving donkey owners, farriers, veterinarians and researchers. Faisalabad is an industrial city in the Punjab province of Pakistan, where donkeys are used for goods transportation. Working donkeys require regular farriery to avoid hoof and limb problems, including lameness. The perception of owners regarding the farriery needs of their donkeys, and the farrier’s understanding of quality farriery, play an important part in ensuring donkey welfare. In this study, owners were asked questions about lameness issues in their donkeys, farriery intervals, factors that they considered important in choosing a farrier, as well as problems that they experienced with farriery. Farriers were asked about their business structure, challenges, and the farriery requirements of donkeys in the region. Farriers reported a lack of training, meager wages, and that farriery is an unregulated industry in Pakistan. Owners provided mixed reports regarding the quality of farriery their animals received. Owners ranked the relationship with their farrier as the most important consideration when selecting a farrier, with cost not being a major factor. This study provides important information for interventional projects taking place in the region for the improvement of donkey welfare, and mitigation of lameness and foot-related problems.

**Abstract:**

Quality farriery is essential to ensure donkey welfare, and many intervention programs in low-middle income countries (LMICs) train farriers, and educate owners, regarding the farriery needs of donkeys. It is essential for interventional programs to understand the perspectives of all stakeholders in donkey-owning communities. A cross-sectional questionnaire study was conducted in the Faisalabad region of Pakistan. Ten farriers and 55 donkey owners participated in the study. Farriers reported receiving no formal training for farriery, insufficient wages (PKR 65 or USD 0.36 per hoof) and the requirement to use traditional tools. Farriers reported an average shoeing time of 5 min per hoof and shoeing between three and 30 donkeys a day. Donkeys were mostly shod while they were harnessed to a cart. Six donkey-owning farriers reported shoeing their donkeys every 20 to 25 days. Owners reported varying shoeing intervals of 20 days to 90 days. Shoeing interval was also affected by seasonal and weather changes. Owners considered their relationship with farriers as the most important factor in choosing a farrier rather than cost. A majority (87%) of the owners reported lameness in their donkeys. The study provides important insights into the many challenges and opportunities in developing quality donkey farriery in the region.

## 1. Introduction

Equines are an essential part of the lives of small-holder farmers in low-middle income countries (LMICs) [1] and are recognized as an important source of transport, and for moving agricultural and other goods including construction materials, such as bricks in brick kilns [2]. There are 117.5 million equines worldwide, of which 50.6 million are donkeys [3]. In LMICs, most donkeys are kept as working animals [4], but demographics are changing with increased mechanization and shifts in demand for donkey-derived meat, milk and hide products in some regions [5,6]. However, the economic role of working equids is often under-estimated, or not assessed by policymakers [7].

Pakistan is a LMIC [8] and is home to 6 million equines of which 5.4 million are donkeys [3], with many classified as draft animals. The efficient use of working animals is dependent upon their management and husbandry [9]. However, the welfare of working equids may be limited by the knowledge and resources of their owners or keepers [10], with many owners being illiterate, and the animals themselves having little or no monetary value [11]. These limitations do not support or facilitate owner access to recent advances in equine husbandry and give rise to many welfare and production-related problems [12].

Farriers and farriery practices play a crucial role in equine husbandry. Good hoof care is a result of collaboration between farrier, veterinarian and equine owner [13]. In the donkey, many hoof problems, such as laminitis, white line disease and flexural deformities have been directly linked to poor farriery practices [14]. In working donkeys in Pakistan, the incidence of lameness was between 90–96% [4,15]. Additionally, it was identified that 90% of the lame animals had a hoof or limb problem [16], and almost all lameness cases could be linked to poor farriery practices. In working horses, hoof neglect has also been linked to negative animal behavior and lower body condition score (BCS) [17].

Farriery practices in LMICs, including Pakistan, have been poorly developed [18]. However, the World Organisation for Animal Health (OIE) suggests that ‘Hoof trimming and shoeing of working equids should only be performed by persons with the necessary knowledge and skills’ [2]; lack of training may present a barrier to meeting this goal. Owner education also plays a crucial role in improving welfare, as initiatives have greater success if education was combined with veterinary care, rather than veterinary care alone [19]. For example, a participatory intervention in Jaipur, India, used regular community meetings facilitated by a trained community member to raise awareness of common husbandry issues and developed community awareness and goals. This resulted in a significant improvement in lameness in horses over a two-year period [20].

The Faisalabad Division, a region of Pakistan, has a large equine population. While studies have been conducted in Faisalabad investigating disease burdens (e.g., [21,22,23], to date, no studies have quantified the husbandry and management of equids with particular reference to the farriery practices that are available for working equids in the region. There is a need to properly understand the owner-equid relationship in Pakistan so that equine welfare strategies can be constructed and implemented effectively.

The objectives of the current study were to identify common farriery and hoof care practices undertaken by farriers and to identify factors affecting owner decision-making in choosing farriery services. By identifying current practices, and engaging with the community through research, good practices can be identified and relationships built. As such, this study will form the foundation before addressing behavioral changes or educational needs by farriers and donkey owners, in relation to working equid hoof care.

## 2. Materials and Methods

### 2.1. Study Population

Data were collected from different donkey-owning communities and farrier workplaces in the Faisalabad district of Pakistan from February to June in 2020, using a convenience sampling technique. Data were collected over the winter and in the summer seasons. Data were not collected during the spring season due to the nationwide lockdown in Pakistan to prevent SARS-CoV-2 (COVID-19) spread. The study comprised a cross-sectional questionnaire of farriers and donkey owners, including farriers who also owned their own donkeys, in order to develop a deeper understanding of a farrier’s point of view of animal welfare.

All the interviewed owners and farriers belonged to the Faisalabad city area. The farriers did not have shops but rather worked on the roadside. The farriers were easily spotted on roadsides and rapport was built by the researcher before conducting the interview. The farriers were generally illiterate, and therefore the purpose of the interview had to be clarified verbally to each individual. This removed any concern that they were being assessed by government officials or tax collectors, and therefore, they answered to the best of their knowledge. Owners were recruited by approaching clients of the farriers in the same areas, with the permission of the farriers.

Surveys were recorded on an offline, bespoke, Android application named “Lameness Survey” specifically designed for this project. The responses were recorded by the interviewer (RZUK) in Urdu and later translated to English before data analysis was performed. The data were synchronized once the internet was available and was then exported in .csv format for analysis. The link to the database of the software has been password protected for security purposes and to avoid data tampering. All participants were invited to participate in the interview and verbal consent was obtained.

The owner questionnaire was structured to consist of 13 multichoice or yes/no questions, one ranking question and two open-ended questions. The farrier questionnaire was structured and consisted of 20 multichoice or yes/no questions and two open-ended questions. Terms such as ‘lameness’ were explained by the interviewer when needed. Questionnaire questions may be found in Supplementary File S1. The whole interview process took between twenty to thirty minutes. The questionnaire was pilot tested with two participants in a neighboring region. No changes were made following the pilot, but these responses were not included in the final study.

### 2.2. Data Analysis

Data were exported from the Android application and analyzed in Microsoft Excel (2016, Redmond, WA, United States). Continuous data were non-normally distributed and were presented as medians and ranges. In some cases, partially continuous data required categorization and were presented as numbers and percentages. Categorical data were presented as a count and percentage. Figures were generated as appropriate.

The open-ended question regarding the frequency of lameness was treated as qualitative data, with a summary by frequency category based on identifying keywords in the owner’s response. Similarly, the improvement in donkey gait following farriery was coded as a binary yes or no. For both questions, the number and percentage of owners in each category were presented and direct quotes were provided where appropriate. To ensure anonymity, owners and farriers were identified by a number and direct quotes provided in italics. If changes to the original quote were made by the authors to enhance clarity, these are presented in square brackets and are not italicized.

The project was approved by the City University Human Ethics Sub-Committee, reference number H002176. Participation was voluntary and data were anonymized in a password-protected database.

## 3. Results

### 3.1. Farriers

#### Farriers as Business Owners

There were ten farriers who answered the farriery business questions. Six farriers were based at a set location and four conducted their business at different locations. Eight farriers shod equines—donkeys, horses and mules. Two farriers also provided trimming services to dairy animals.

Of the ten farriers interviewed, five inherited the business, four started the business themselves and one reported a mixture of inheritance and self-start up. Seven farriers noted they had learned their trade from their relatives, while three learned from a person who was considered to be a senior or experienced farrier. Two farriers noted that training provided by a non-government organization (Brooke Action for Working Horses and Donkeys) was a part of their farriery training. Six farriers then said that they would only seek to give the business to their children if there was no other option, with nine citing low wages as the main issue with the profession. Two farriers said that the harsh environment or weather conditions (*n* = 2) made their work difficult, with the temperature ranging from 10 °C in winters to 45 °C in summer. No farriers had clean drinking water available at their work sites. Other issues were the unpredictable market (*n* = 1) and disrespect for the profession (*n* = 1). All the farriers lacked safety gear for injury protection and disease protection due to unaffordability. Six farriers noted that they had experienced a workplace injury. Five farriers had been kicked, one farrier had been cut with their tools and one farrier had been trampled. The severity of these injuries was not disclosed.

Farriers reported that in all cases the animal’s owner would determine when it was shod (*n* = 10), four of those in consultation with the farrier. One owner sought advice on when to shoe from the farrier and veterinarian. Eight farriers noted that they would provide advice to owners and that the farriers were also the first point of contact regarding any kind of illness in equines.

In terms of the day-to-day farriery business, farriers reported shoeing between three and 10 donkeys (*n* = 5) or 20 to 30 donkeys (*n* = 4). One farrier reported shoeing between five and 20 donkeys per day. Donkeys were reported to take 2 to 3 min (*n* = 2), 5 min (*n* = 7), or 5 to 10 min (*n* = 1) per hoof to shoe, with shoeing occurring while the donkey was still in the harness. Per hoof, farriers charged PKR50 to 60 (*n* = 4), PKR 65 to 75 (*n* = 5) or PKR80. This represents a range of 0.28 to 0.45 USD per foot or 1.13 to 1.81 USD per donkey.

**Figure 1:**
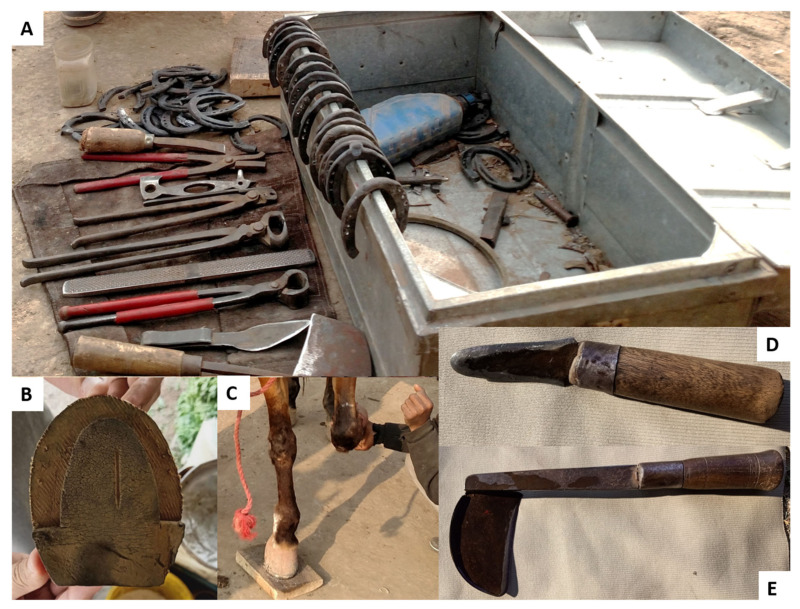
A typical farrier workstation (**A**) with examples of tools and iron shoes. (**B**) Rubber shoe insert (**C**) Plank being used to check hoof balance (**D**) Hoof knife (**E**) ‘Traditional’ hoof knife.

All farriers used shoes and nails within their business. Seven farriers only used iron shoes. The remaining farriers used iron and rubber (*n* = 3). All farriers purchased some of their equipment, with two farriers producing equine shoes in their own furnaces from iron construction rods, whereas the rest of the farriers purchased shoes from hardware shops. All farriers reported using the traditional hoof knife (Figure 1E), a hoof knife (Figure 1D), a hoof or flat rasp, and an anvil. Nine farriers reported using a rounding hammer, a driving hammer, and hoof nippers. Eight farriers reported using a flat wooden plank to check hoof balance, seven reported using nail nippers, six used a chisel, four used clench cutters and four used a hoof pick (Figure 1). Two farriers made some of their own tools. Many tools were partially worn out, with only the blades in working condition. Several owners and farriers reported injuries caused by worn grips or handles.

### 3.2. Farriers Who Owned Donkeys

Six of the farriers owned one donkey and one farrier owned two donkeys. Farrier-owned donkeys were kept for transportation to work. Some farriers were also equine dealers and traders, depending upon market demands. Five farriers provided hoof care for their own donkeys every 20 to 25 days; one provided farriery every 25 to 30 days. Five farriers adjusted the frequency of shoeing seasonally, with one farrier noting that the frequency was adjusted based on the weather.

### 3.3. Donkey Owners

Fifty-five donkey owners participated in the study. In total, 43 owners had one donkey, nine owners had two donkeys and three owners had three donkeys, for a total of 67 donkeys (median 1, range 1 to 3 donkeys). Eight donkey owners also owned goats (*n* = 2), hens (*n* = 2), dogs (*n* = 3), and a cow (*n* = 1).

Forty owners (73%) sought farriery services every 15 to 30 days, 11 (20%) every 30–60 days, and four (7%) sought services every 2 months or more (up to 3 months). One owner did not respond regarding the time between hoof care occasions in different seasons and three owners did not know. Nineteen (35%) owners said that they did not adjust their farriery regimen seasonally, while 32 (59%) did. Four owners noted the reason that they adjusted the timing of shoeing was to do with the weather, or changes in heat or dampness, while one owner included working conditions as a consideration.

**Figure 2:**
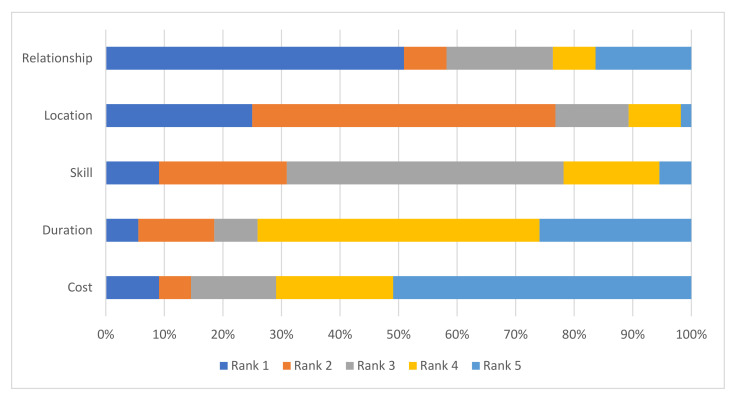
Owner’s ranking of the reason that they selected a particular farrier to provide services to their donkey. Relationship—the relationship between the owner and the farrier; Location—the location of the farrier; Skill—the skill or knowledge of the farrier; Duration—the time between shoeings; Cost—the charge per hoof.

In total, 43 (78%) owners always used the same farrier. The median cost per foot for shoeing was PKR 65 (range PKR 55 to 80; USD 0.31 to 0.46) as reported by owners. Seven owners reported that their farrier assessed their donkey for lameness at some point before or after shoeing. Owners ranked their own relationship with the farrier (as a friend or relation) (*n* = 28; 51%) as most important for farrier selection, followed by the farriers location (*n* = 14; 26%), the cost (*n* = 5; 9%), the farrier’s skills or knowledge (*n* = 5; 9%) or the time between shoeings (*n* = 3; 5%) (Figure 2). The second ranking included location (*n* = 29; 53%), the farrier’s skill or knowledge (*n* = 12; 23%), time between shoeings (*n* = 7; 13%), relationship with the farrier (*n* = 4; 7%) or cost (*n* = 1; 2%). The third ranking included the farrier’s skill or knowledge (*n* = 26; 47%), relationship with the farrier (*n* = 10; 18%), cost (*n* = 8; 15%), location (*n* = 7; 13%), and time between shoeings (*n* = 4; 7%).

**Figure 3:**
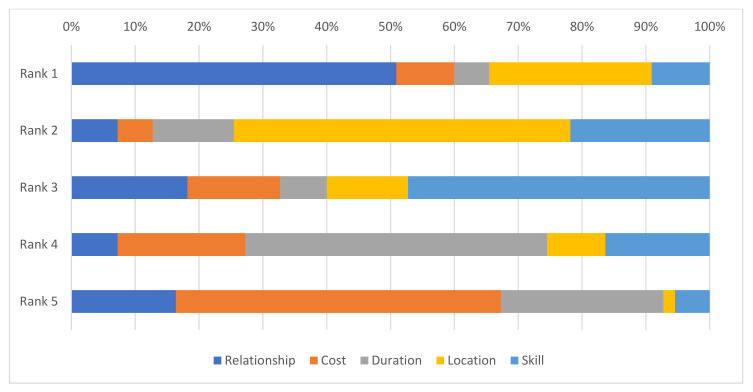
Owners ranking of the reason that they selected a particular farrier to provide services to their donkey. Relationship—the relationship between the owner and the farrier; Location—the location of the farrier; Skill—the skill or knowledge of the farrier; Duration—the time between shoeings; Cost—the charge per hoof.

If relationship had the highest rank, the second rank was location (*n* = 25/28), the third rank was the skill or knowledge of the farrier (*n* = 25/25), the fourth rank was the time between shoeings (*n* = 22/25) and the fifth rank the cost of shoeing (*n* = 22/22) (Figure 3). If location had the highest rank, the second rank was the farrier’s skill or knowledge (*n* = 6/14), the third rank the relationship with the farrier (*n* = 5/6), the fourth rank was cost (*n* = 4/5) and the fifth rank the time between shoeings (*n* = 4/4).

### 3.4. Donkey-Owner Reported Lameness

Owners discussed the frequency of lameness, and whether farriery changed the status of lameness in their donkeys. In total, 17 (31%) owners noted that their donkey(s) were always lame, the majority of owners who reported that their donkey was always lame described this as slight (*n* = 9), for example, ‘*slight lameness exists almost all the time*’ Owner 14. More severe lameness was described less frequently (*n* = 7), with one owner describing the situation as ‘*the animal is continuously lame and in pain*’ Owner 29. Eighteen (33%) owners noted that their donkey was intermittently lame, for example, ‘*the animal usually gets lame once a year at least*’ Owner 45. Five owners discussed their animals becoming lame in response to seasonal or climatic conditions, for example, ‘*the animal is usually lame in harsh weather conditions*’ Owner 33, with some owners specifically noting the season ‘*it usually gets lame in winter*’ Owner 28. Thirteen owners (24%) reported their donkey becoming lame following an event. One owner said, ‘*the donkey gets lame after accidents and once when it got overloaded*’ Owner 26, while Owner 4 described a particular type of event and the frequency: ‘*It depends upon slipping* [falling] *and that can occur 2–4 times in a year*’. Some owners noted that lameness following events could occur several times per year but was not seasonal or predictable. Other owners talked about these events occurring, but it may have occurred on one occasion while they had owned the donkey, rather than being a more frequent occurrence. With regards to lameness occurrence, farriery practices were mentioned by five owners. Owner 48 commented ‘*the animal went into non-weight bearing lameness, but as soon as the farrier trimmed the hoof, the animal started to put some weight on hoof*’. Some owners did not have such positive stories, for example, ‘*the farrier cut the frog and since then the animal is lame*’ (Owner 36) and ‘*it got lame once during farriery* [from a] *nail prick*’ (Owner 27). Seven owners (13%) reported never having a lame donkey. With regards to the effect of farriery on lameness, 29 owners (52%) described farriery improving lameness, for example, ‘[the] *gait improves and no lameness caused by* farriery’ Owner 21. Twelve owners (22%) reported no resolution of lameness, with Owner 36 saying ‘*no improvement and* […] *aggravates the situation instead of* [being a] *treatment*’. Fourteen owners (25%) noted that farriery had been the cause of lameness on one or more occasions. Owner 11 replied ‘*yes it gets better. But once in a while it becomes worse*’ with other owners noting the reasons for lameness following farriery being over trimming, nail pricks and Owner 6 noted ‘*bleeding once or twice during farriery*’.

## 4. Discussion

The current study describes the farriery practices and owner perceptions of farriery services in the Faisalabad region of Pakistan. The study enabled an exploration of farriery practices in order to develop a deeper understanding of a farrier’s view of shoeing working donkeys, and barriers within the practice of farriery in this region. In addition, donkey owners were asked to describe lameness in their donkeys and how this related to farriery practices. Owners reported that farriery could have a positive or negative effect on donkey lameness and that around half of their donkeys were lame, demonstrating an understanding of the links between farriery and lameness. We suggest that an improved understanding of farriery practices, linked to appropriate participatory interventions, could have a positive impact on welfare and productivity.

In the current study, the farrier-reported fee for shoeing one hoof ranged from USD 0.28 to 0.45, which corresponds to 1.13 to 1.81 USD per donkey for services, for up to 30 donkeys per day. The charge for farriery services included the cost to the farrier: the cost of the shoe, nails, and the supply and depreciation of tools. Additionally, the fee per hoof would cover daily expenses, for example, travel to work, and include provision should a workplace injury occur, as there is currently no centralized service in Pakistan that would support an injured self-employed worker; however, this is likely to change with the advent of universal government-sponsored healthcare.

Farriery is a very low-paying job in LMICs, such as Pakistan, with low profit margins [24]. While farriery was considered to be a family business, many farriers expressed a reluctance to pass the business on to the next generation, citing low wages as a major issue. A previous study identified that profit margins are also low in India, leading to many farriers developing alternative income streams [24]. This was also seen in Faisalabad, with two farriers selling donkeys and providing hoof trimming services for dairy cattle. This is likely to be attractive because in Pakistan, the dairy sector is growing rapidly [25]. While the impact of the growth of the dairy sector and farriers servicing both sectors were not specifically investigated in the current study, the overlap does create some concerns for the working equid industry. Firstly, the characteristics of a functional working donkey hoof and that of a dairy animal differ markedly [26,27]. A lack of understanding of the similarity or differences in hoof characteristics of these species could be detrimental to welfare. Secondly, dairy animal farriery may be more attractive for the farriers because of the higher number of animals at the dairy farm, and the associated ability to make more money on any given day. Additionally, the working environment in a dairy farm is more favorable, with work in a barn with climate control and improved animal restraint. This could lead to a lack of farriers available or willing to provide services for donkeys, with potential implications for donkey welfare.

In the current study, owners have reported that the shoeing interval varies from every 15 to 30 days (73%), every 30 days to up to 2 months (20%), and 7% of owners sought services every 2 months or more (up to 3 months). The majority of owners reported a shoeing interval within recommended interval ranges [28]. All farriers who owned donkeys shod their donkeys more frequently and reported the range to be between 20–30 days. It should also be kept in mind that the shoeing interval requirements can be different than recommendations devised in other regions of the world because of the nature of work of the animals, the difference in terrains and the quality of shoes and shoeing practices. It was also observed that farriers and owners changed shoeing frequency according to the weather. Five owners also discussed their animals becoming lame in response to seasonal or climatic conditions. It has been reported previously that there is a difference in frequency of specific lameness reports in horses with the change in season [29], and that white line abscessation is more common in donkeys in the UK in wet weather [30]. There is currently a lack of data on how weather affects optimal shoeing intervals in shod donkeys.

The majority of farriers reported that shoeing took less than five minutes per hoof, with shoeing being carried out while the donkey was still in the harness. It is generally considered that proper shoeing and trimming require the farrier to observe the donkey from a distance and perform a physical examination as well as evaluation of shoeing and hoof balance [31]. This is likely to be challenging with the donkey harnessed and requires more than five minutes per hoof. Therefore, the time devoted to farriery was found to be less than required for acceptable quality of delivery.

“This is where the rubber meets the road; if we cannot make it pay, we cannot spend as much time to it as required for quality farriery”.

[31] (p. 277)

This problem is complex. Low pay reduces the time spent on farriery, likely leading to a reduced willingness for owners to pay a fee that would cover the additional time required for higher quality farriery. This is where teamwork and engaging all stakeholders is important, as there is a possibility that a flourishing owner–farrier relationship can bring improvement for donkeys.

In our study, two farriers reported receiving training from a non-government organisation (NGO) operating in the area, while most farriers learned their trade ‘on the job’ and from people who were already working as farriers. It is recognized that an informal workforce with few or no regulations can lead to poor-quality farriery [24], and in Pakistan, farriery is an unregulated industry, and there are no training institutions to build the capacity of the workforce [18]. However, NGO farriery projects can yield great success. In a pilot study in Ethiopia, improved farriery and training practices were trialed on 33 Garri horses with two farriers, where success measures including foot health indicators and improved welfare in the horses were identified [32]. Additionally, and as a secondary measure of the project′s success, owners not involved in the project requested improved farriery services despite higher prices. Subsequently, other farriers requested training, thus improving the overall quality of farriery services. It has also been shown in other studies that equine owners seek quality farriery, even at a higher cost [33]. Similar outcomes have been reported in horses in Sudan (R. Hovell, personal communication). In the current study, the impact of farrier training was not reflected in practice, with the exception of shoeing interval.

In the present study, when owners were asked about their considerations for selecting a farrier, cost did not rank highly. While location was ranked as important, care should be taken in interpreting this result, as donkey owners were selected by virtue of attending a farrier who agreed to participate in the study, at the time of the farrier interview. Despite this, the owner–farrier relationship and the skill of the farrier were considered highly important. In contrast, cost and time were perceived as highly important by farriers in a study of working equids in India, but farrier relationship [to the owner] and reputation were also recognized as important [20]. One example of the importance of the owner–farrier interaction is the practice of over-trimming or removal of the frog, which is frequently requested by donkey owners in Pakistan and has been reported in India [20]. This illustrates that there is scope to improve welfare for working donkeys, through engagement with farriers and providing evidence-based, practical solutions to improve farriery practices, in addition to improving the status of farriers as experts in their field. Specifically, a good owner–farrier relationship is needed so that owners will respect the opinion of farriers as experts if they refuse to undertake harmful practices, such as frog removal. One limitation in providing training may be a reliance on reference values for donkey hoof morphometry from horses or from breeds from other regions of the world rather than from local breeds [26].

Another area for improvement in the profession is demonstrating that quality farriery can outweigh the cost of poor farriery by reducing treatment costs for lameness, reducing lost workdays, and potentially extending the shoeing interval. This may also help to address the concerns of farriers that donkey farriery is a profession with limited prospects in LMICs; if farriers felt that a better living were to be made, perhaps this would ameliorate concerns about passing on the profession to their children. However, increasing the cost of farriery services to provide a better living may be challenging, as farrier income is linked with the income made by owners through their donkeys. While the daily earnings of donkeys in Pakistan were between USD $2.50 and $5.00 per day [34], it is unlikely farriers will see the significant financial benefit until the utility of donkeys is financially recognized.

Farriers in the study often use trimming and shoeing tools and techniques that would be considered ‘conventional’ in Europe or the USA but are performed with tools that are used traditionally in Pakistan. However, many tools were partially worn out, with only the functional parts (i.e., blades) in working condition. The tools sometimes lacked proper grips and had been reported by owners and farriers to have caused injuries to both donkeys and farriers by allowing the blade to slip. Overused tools and lack of modern farriery techniques could be due to the low income of the farriers; a low-income profession does not invite investment in training and may not be able to afford investment in quality tools. Farriers do not have to pay rent for their spots on roadsides, and therefore, they can be moved on by government officials at any time, which could lead to a lack of security.

The majority of donkey owners reported lameness in their donkeys. Only seven owners did not report lameness at any time, which is consistent with a recent study in this population [26]. While not common, owners in the current study also reported their donkeys becoming lame due to traumatic events, such as slipping, being hit by a vehicle, or overloading. These issues have been reported elsewhere, although currently, no good definition of overloading exists for donkeys [35]. Overall, owner recognition of lameness is poor worldwide, with 47–75% of sports horses [36,37] and 60–67% of polo ponies [38] considered sound by their owners in fact exhibiting lameness, gait abnormalities, or movement asymmetries outside of ‘normal’ ranges. Lameness in working donkeys is much higher than in these populations [1,8]. Moreover, lameness detection in donkeys is difficult because they are stoic in nature and tend to hide pain [39,40]. This further indicates the importance of developing a donkey-specific lameness evaluation system that could help owners in the early diagnosis and treatment of lameness in their donkeys. Modified lameness evaluation scales [26], grimace scales [40] and ethograms [41] represent important aids in this respect, but knowledge of these tools needs to be transferred to owners and farriers along with veterinarians working with working donkeys.

In the current study, 52% of owners commented that farriery practices improved donkey soundness by reducing lameness. Some owners also noted that farriery on occasion made lameness worse, by over-trimming, mutilation of the frog and ‘nail prick’. Mutilation of the frog can be one of the factors that hinders the expansion of the equine foot in the landing phase of the gait in horses [42], but little is known about ‘normal’ donkey gaits or hoof-ground interactions. Improved education for both owners and farriers is critical, but these interventions need to be participatory and inclusive [20,24].

It is important to understand that every community of equine owners faces different social norms, economic constraints, and perceptions of the welfare of working animals. Therefore, a regional understanding of these factors can yield more fruitful outcomes when translated to interventions [33,43]. The current study is important because it involved local veterinarians for study design, data collection and interpretation. This approach removes many limitations in terms of potentially culturally sensitive matters, ensuring discussions were held in the local language, fear of disclosing local practices to outsiders and removing any response bias from trying to feed desirable information to outsiders for gifts and rewards. This study is limited by convenience sampling of the survey population and the use of closed questions. Further work with focus groups or with a predominantly qualitative approach would add more context and allow a deeper understanding of the local issues surrounding farriery and hoof care in working donkeys in Pakistan.

## 5. Conclusions

These findings are one step towards understanding the owner-equid and health management of working equids in Pakistan, facilitating the development and implementation of effective equine welfare strategies. Additionally, this study forms the basis of developing and maintaining relationships between researchers and the community, to help improve animal welfare and productivity by improving hoof care in working equids.

## Data Availability

Due to data privacy concerns and conditions of the verbal consent given by owners, the full dataset is not publicly available. Queries can be made to the corresponding author.

## Supplementary Materials

The following supporting information can be downloaded at: https://www.mdpi.com/article/10.3390/ani12060709/s1. Farrier questionnaire, owner questionnaire.
