# Supplementary material for: Cross-Sectional Questionnaire of Donkey Owners and Farriers Regarding Farriery Practices in the Faisalabad Region of Pakistan
Source: Animals (Basel). 2022 Mar 11;12(6):709. doi: 10.3390/ani12060709 (PMC8944430; doi:10.3390/ani12060709)
Supplement: Supplementary file 1 [file animals-12-00709-s001.zip › animals-1577851-supplementary.pdf]

animals

MDPI

Supplementary File S1

The questions below were used by the interviewer and andwers recorded on an offline, bespoke, Android application named "Lameness Survey" specifically designed for this project. Interviews were conducted verbally, and questions were translated into Urdu. Any terminology was explained where necessary.

Farrier Interview

1. Gender
2. Date
3. How many horses/donkeys do you shoe per day?
4. How much do you charge for shoeing (per hoof)?
5. Which tools/ equipment are present in your kit?
   (at this point, photos could be taken with the farrier's consent to illustrate tool types).
6. Do you make shoes yourself, or do you purchase them?
7. Have you ever sustained any work-related injuries, and if so, what types?
8. Which individual has the most input into the shoeing?
   Farrier
   Veterinarian
   Owner
9. Do owners bring their animals or shoeing regularly?
10. What is the interval between shoeing?
11. How frequently do your tools need replacing?
12. How much time does it take you to perform one standard shoeing?
13. Do you keep any medicine in your kit?
14. Do owners visit you for advice when animal has farriery/ lameness issues?
15. Which animals do you shoe? (equids only or dairy animals too)?
16. Which material do you use to make shoes (steel or rubber)?
17. Do you work at a particular place/shop or do you visit other places for farriery?
18. How did you learn to be a farrier?
19. How did you develop your business? – e.g., did you inherit it, start it yourself etc.
20. What difficulties does your business face?
21. Would you transfer this business to your children?

Owner Interview

1. Gender
2. Date
3. How many animals do you have?
4. How often do you take your donkeys to see the farrier, and does this vary depending on season?
5. Do you always use the same farrier?
6. How much do you pay for shoeing (per hoof)?
7. Please rank these factors in order of importance regarding how you choose your farrier:
   a) Cost
   b) Location
   c) How long the shoe lasts between two shoeings
   d) The farrier's skill/knowledge

e) Your own relationship with the farrier/how you know him (e.g., relative, friend etc)

8. Have you ever observed lameness in your donkey, and if so how frequently?
9. After shoeing, does your donkey's gait improve? Has your donkey ever been lamed following farriery?
10. When you see any lameness in your donkey, do you visit your farrier?
11. Is this your first farrier or do you switch between farriers?
12. Does your farrier assess your donkey before and after shoeing?
